# Sex-Specific Pharmacotherapy for Migraine: A Narrative Review

**DOI:** 10.3389/fnins.2020.00222

**Published:** 2020-03-20

**Authors:** Parisa Gazerani, Brian E. Cairns

**Affiliations:** ^1^Laboratory of Molecular Pharmacology, Department of Health Science and Technology, Faculty of Medicine, Aalborg University, Aalborg, Denmark; ^2^Faculty of Pharmaceutical Sciences, The University of British Columbia, Vancouver, BC, Canada

**Keywords:** migraine, headache, female, male, sex, prophylactic, acute, abortive

## Abstract

Migraine is a common neurological disorder characterized by recurrent headache episodes that accompany sensory-motor disturbances, such as higher sensitivity to touch and light, extremity heaviness or weakness, and speech or language disabilities. Worldwide, migraine is one of the top 10 causes of disability and hence poses a huge economic burden to society. On average, migraine occurs in 12% of population but its occurrence is sexually dimorphic, as it is two to three times more prevalent in women than in men. This female to male ratio of migraine prevalence is age- and sex hormone-dependent. Advancements in understanding migraine pathogenesis have also revealed an association with both genetics and epigenetics. The severity of migraine, in terms of its attack duration, headache intensity, frequency, and occurrence of migraine-associated symptoms, has generally been reported to be greater in women. Sex differences in migraine disability and comorbidities, such as psychiatric disorders, have also been noted in some population-based studies. However, research on sex-related differences in response to migraine treatments is relatively scarce. Although a general observation is that women consume more medication than men for migraine treatment, strategies for the use of abortive and preventive medications for migraine are generally similar in both sexes. This narrative review summarizes available findings on sexually distinct responses to abortive and prophylactic pharmacotherapy of migraine. Basic experimental data and clinical findings will be presented, and potential mechanisms underlying sex-based responses will be discussed to highlight the importance and value of sex-based treatment in migraine research and practice.

## Narrative Review

This narrative review provides an overview of the current knowledge on sex-specific pharmacotherapy for migraine with the idea of summarizing available information in the field and highlighting pending questions that are yet to be considered in future studies. As pointed out carefully by Peterlin et al. (2011), recognition of factors influencing sex-based responses in migraine is important; however, it needs to be followed with utilization of findings in a meaningful manner both in migraine research and practice to advance treatment and prevention of this disorder.

Migraines are moderate to severe primary headaches that may be preceded by aura, and accompanied by nausea and photophobia. The prevalence of migraine is similar in pre-pubescent boys and girls, but beginning at puberty, young women suffer twice as commonly from migraine as young men (Finocchi and Strada, 2014; Wilcox et al., 2018). In women, the peak prevalence of migraine occurs around age 30–40 years, after which it declines (Finocchi and Strada, 2014). Women also suffer more attacks per month than young men of the same age, and about twice as many will progress to a chronic form of the headache (>15 headaches per month) (Wilcox et al., 2018). Women have longer, more intense headaches and a greater overall sensory hypersensitivity, particularly facial cutaneous allodynia (Finocchi and Strada, 2014; Wilcox et al., 2018).

It is thought that sensitization of dural afferent fibers causes the head pain symptoms typically reported by migraine sufferers (Levy et al., 2018). Neurogenic inflammation, which results from a localized release of neurotransmitters, such as serotonin, histamine, and glutamate as well as neuropeptides, such as calcitonin gene-related peptide (CGRP) and substance P, may underlie this sensitization. These substances produce alterations in cerebral vascular tone, promote plasma protein extravasation, and decrease the activation threshold for dural afferent fibers. There is still controversy over how the process of neurogenic inflammation is triggered. Given the diversity of migraine headache triggers in patients, it is likely that both central and peripheral mechanisms are involved.

Estrogen appears to play a role in sex differences. Dural application of “inflammatory soup” (IS) has been used to examine behavioral or electrophysiological changes associated with meningeal inflammation. Females show an increased sensitivity to IS application compared with males (Stucky et al., 2011). Elevated estrogen levels increase the response of dural afferent fibers and brainstem trigeminal sensory neurons (Bolay et al., 2011; Scheff and Gold, 2011; Finocchi and Strada, 2014; Pavlovic et al., 2017). Both estrogen receptors (ERs), ER_α_ and ER_β_, are widely expressed by trigeminal ganglion neurons (Wang et al., 2012; Pavlovic et al., 2017). Estrogen can exert a direct sensitizing effect on trigeminal afferent fibers specifically through ER_α_ (Rowan et al., 2014). Women who suffer migraine with aura have elevated plasma estrogen levels compared with women who have migraine without aura (Nagel-Leiby et al., 1990). Migraine without aura is often reduced during pregnancy, whereas migraine with aura is often worsened by oral contraceptive use (Bolay et al., 2011). It is hypothesized that the rate of change of estrogen levels is a trigger for headaches, for example, women with migraine appear to have a faster decline in estrogen prior to headache than healthy women (Macgregor et al., 2006; Pavlovic et al., 2017). Men with migraine have also been reported to have higher plasma levels of estrogen than men without migraine (Van Oosterhout et al., 2018). Other hormones, such as testosterone, progesterone, and prolactin, as well as genetic and epigenetic factors, may contribute to these sex-related differences in migraine (Gazerani and Vinterhøj, 2016; Delaruelle et al., 2018; Gazerani, 2019).

Sex-related responses in migraine are not limited to biological factors, such as sex hormones. Genetic and epigenetic factors, environmental stressors, psychosocial factors, and coping strategies in response to stress and pain perception are among other contributing factors. The magnitude and influence of psychosocial factors (including stress, anxiety, and irritability) have been found greater in female migrainures than in males (Lebedeva et al., 2017). Attempts have provided evidence on psychosocial considerations in migraine. Psychological interventions have also been proposed to offer beneficial value to pharmacotherapy (Sullivan et al., 2016). Readers are referred to other reviews in the field (e.g., Raggi et al., 2012). Migraine is also co-morbid with several disorders, such as psychiatric disorders and comorbidity not only influences the course and severity of migraine but also responsiveness to treatment and prevention.

### Sex-Related Differences in Response to Pharmacotherapy

The pharmacotherapy of migraine involves two strategies: treatment of the acute headache with drugs intended to reduce or eliminate headache pain, termed abortives, and use of drugs intended to prevent migraine headaches, known as prophylactics. The response to abortive agents is usually assessed at 2 and 24 h after drug administration. It is often described in the literature as the percentage of sufferers who are “headache free” at these two time points, however, studies also report the number of subjects who have a clinically meaningful reduction in their headache pain, which varies from a 30 to 50% decrease in headache pain intensity, depending on the authors. The efficacy of prophylactic therapy is commonly assessed by a reduction in the number of days with headache, or alternatively the number of headaches. Many studies consider that a 50% reduction in headache days is a clinically meaningful measure of the efficacy of prophylactic drugs. As most studies rely on sufferer-reported data, there is significant interest in developing biomarkers to more objectively monitor the efficacy of migraine pharmacotherapy; however, a reliable, commonly accepted biomarker for migraine treatment has not been identified (Gazerani, 2019). One promising approach is to combine genetic and brain imaging markers to identify the optimal migraine therapy (Nyholt et al., 2017).

#### Abortive Treatment of Migraine

Abortive agents can be classified by their pharmacological action into the following groups: Ergot alkaloids, triptans, non-steroidal anti-inflammatory drugs (NSAIDs) and paracetamol, opioids, and other miscellaneous agents. Studies rarely look at sex-related differences in the efficacy of abortives (van Casteren et al., 2019). This omission is perhaps because of the predominance of migraine in women, which results in far fewer men being included in drug efficacy studies for this condition (Vetvik and Macgregor, 2017). Whereas women tend to use prescription medication (e.g., triptans and ergotamine) for abortive medication, men are more likely to use over the counter medications, including NSAIDs, in particular acetylsalicylic acid (ASA), ibuprofen, naproxen, and diclofenac (Buse et al., 2013; Vetvik and Macgregor, 2017). Interestingly, men are less likely to report an adequate response to therapy than women at both 2 and 24 h (Lipton et al., 2016a, b). In contrast, female sex is a significant predictor of recurrence (Dodick et al., 2008). Women may also have a higher relapse rate of migraine headache pain than men, but not all studies support this (Dodick et al., 2008).

##### Triptans

Triptans were developed to more specifically target 5-HT_1__B/D_ receptor mechanisms recognized to be key to the antimigraine mechanism of ergot alkaloids. They are vasoconstrictive, through an effect on serotonin (5-HT)_1__B_ receptors, and suppress release of neuropeptides through 5-HT_1__D_ receptors, which are located on cerebrovascular endothelium and dural afferent fibers, respectively. These actions reduce neurogenic inflammation and dural afferent sensitization. Lasmiditan is a newly approved agonist of the 5-HT_1__F_ receptor. Activation of this receptor decreases neuronal excitability in the migraine headache pain pathway presumably by a central effect, as this drug does not appear to exert the typical peripheral effects of triptans, such as vasoconstriction (Goadsby and Classey, 2003).

In animals, 5-HT levels in the dorsal raphe nucleus are modulated estrogen, through a combination of estrogen-related increased tryptophan hydroxylase levels, and decreased reuptake (Gupta et al., 2011; Aggarwal et al., 2012). As estrogen levels drop, 5-HT levels in the brain decline. Further, estrogen has been found to attenuate 5-HT induced vasoconstriction (Gupta et al., 2011). In ovariectomized female rats, the expression of 5HT_1__B_ receptors in the trigeminal ganglion is reduced by about one-third (Aggarwal et al., 2012). Estrogen treatment of ovariectomized female rats restores the level of 5HT_1__B_ receptor expression to that seen in normally cycling rats (Aggarwal et al., 2012). Injection of sumatriptan is known to induce localized mechanical sensitivity, in a sexually dimorphic manner, with female rats showing sensitivity at lower sumatriptan concentrations than males (Araldi et al., 2017). This effect appears to be mediated by estrogen, as gonadectomy attenuates and estrogen treatment enhances it (Araldi et al., 2017). Somewhat surprisingly, in female rats, sumatriptan induced mechanical sensitivity is mediated through 5-HT_1__D_ receptors, whereas in male rats, it is mediated through 5-HT_1__B_ receptors.

It has been reported that oral administration of frovatriptan 2.5 mg produces peak and area under the curve plasma concentrations that are about twice as high in women as in men due to an apparent lower bioavailability in men (Jhee et al., 2001; Elkind et al., 2004; Negro et al., 2011). Frovatriptan, perhaps due to its long half-life, has a lower rate of headache recurrence than other triptans at 24 h post-administration, but also suffers from a lowered headache relief rating at 2 h (Jhee et al., 2001; Negro et al., 2011). Importantly, naratriptan, rizatriptan, and zolmitriptan also show similar, although less dramatic, sex-related differences in peak and area under the curve plasma concentrations (Ferrari et al., 2011). It is not clear what, if any, relationship exists between the efficacy of these agents in migraine and the plasma concentration achieved (Ferrari et al., 2011; Vetvik and Macgregor, 2017). However, in comparison trials of frovatriptan versus rizatriptan, zolmitriptan, or almotriptan, all produced relatively similar efficacy to ameliorate or eliminate migraine headache, which was not different when men and women were compared (Franconi et al., 2014). A recent meta-analysis did, however, find an increased risk for women to have headache reoccurrence 24 and 48 h after triptan treatment, and concluded that this was not explained by a different initial response to these abortive drugs (van Casteren et al., 2019). This same meta-analysis found that women also have a higher risk of adverse events than men when treated with triptans (van Casteren et al., 2019). Most studies of triptans for migraine have a much smaller number of men than of women and thus response efficacy stratified by sex is not often reported (Vetvik and Macgregor, 2017). This was also the case in the study of Franconi et al. (2014) where the female to male ratio was more than 4:1, and thus it is unclear if this confound prevented the identification of sex-related differences in triptan efficacy (Franconi et al., 2014). Thus, one cannot exclude the possibility that low numbers of men in these studies precluded an identification of an actual sex-related difference in efficacy.

##### NSAIDs

Non-steroidal anti-inflammatory drugs act by inhibition of the enzyme cyclooxygenase, which converts arachidonic acid to prostaglandins, of which prostaglandin E, F_2α_, and I (prostacyclin) are thought to contribute to headache pain, in part by sensitizing afferent fibers and altering the excitability of trigeminovascular neurons (Dong et al., 2009). Paracetamol is a non-specific mild analgesic used by some migraineurs. Its mechanism of action with regard to migraine is uncertain. In a human experimental pain study that examined the effectiveness of ibuprofen 800 mg on electrically evoked ear lobe pain tolerance in healthy men and women, only men showed a significant analgesic response (Walker and Carmody, 1998). The only sex-related difference in pharmacokinetic (PK) parameters was the weight-adjusted volume of distribution, which was twice as high in women. No relationship between plasma ibuprofen concentration and analgesia in men was identified, suggesting that the slightly higher concentrations of ibuprofen found in men were not likely the explanation for this sex-related difference. Subsequent research by the same group found that expectation of analgesia in men was the likely reason for the difference in ibuprofen efficacy in this model (Butcher and Carmody, 2012). The absorption of ketoprofen is more rapid in men than in women, with men achieving higher maximum concentrations in the plasma (Lorier et al., 2016; Magallanes et al., 2016); however, this NSAID is not as commonly used for acute migraine treatment. Not all NSAIDs show a sex-related difference in their PK. Diclofenac potassium oral solution, which is used as an abortive for migraine headache, has not been shown to have a different disposition in men compared with women (Chen et al., 2015). Ketorolac, an NSAID, which is available in injectable form, has been shown to be effective as a migraine abortive agent (Rao et al., 2016). There is no clinical evidence that ketorolac has a sex-related difference in its efficacy (Friedman et al., 2015). However, in an animal model of masticatory muscle pain, peripheral application of ketorolac was found to exert a significantly greater effect in female rats (Cairns et al., 2012). The relevance of this finding to the actions of ketorolac as a migraine abortive is uncertain, but at least theoretically, this suggests that certain NSAIDs may work better in one sex than in another.

Orally ingested ASA is absorbed more quickly in women than in men, but is also hydrolyzed (de-acetylated) and cleared more rapidly from the plasma of women, which may explain a decreased efficacy of ASA as an antithrombolytic in female migraineurs (Buchanan et al., 1983; Jesurum et al., 2012). However, in a retrospective study of 61 migraine patients, no sex-related difference in the efficacy of ASA to ameliorate migraine headache was found, although the authors speculated that plasma concentrations of intact ASA might be predictive of response (Ross-Lee et al., 1983). A study of a combination of paracetamol, ASA, and caffeine (Excedrin) in men and women migraineurs showed a trend toward higher headache pain relief as the percentage of men in the study group was increased (Silberstein et al., 1999). When the study population included 25% men, headache was relieved in 65%, whereas when the study population had only 17% men, headache relief was reduced to only 56% at 2 h post-administration (Lipton et al., 1998). In a study of women alone, Excedrin ameliorated or eliminated migraine in 58% of women at 2 h post dose (Silberstein et al., 1999). Assuming that the efficacy in the study of women alone is representative of the effectiveness of Excedrin, a simple calculation based on the population with the highest percentage of men in the Lipton et al. study can be used to estimate efficacy in men. This calculation suggests that the efficacy of this therapy in men would have been around 86% to produce the overall rate reported in the combined studies. There is some evidence that men are more likely to use ASA for migraine than women, and this might be explained, in part, by an increased efficacy of ASA in men, however, to date, no research has investigated if such a difference exists.

##### CGRP receptor antagonists

The gepants, e.g., rimegepant, are small molecule CGRP receptor antagonists, which have shown some promise as abortives in migraine headache treatment (Croop et al., 2019). Antagonism at this receptor attenuates neurogenic inflammation and is effective in aborting migraine headache (Edvinsson et al., 2018). Evidence, discussed below, suggests that CGRP receptor antagonists could exhibit sex-related differences in their efficacy. In the most recent, placebo controlled study, 85% of migraine patients enrolled were women, and no assessment of sex-related differences in drug efficacy was reported (Croop et al., 2019).

In healthy humans, plasma CGRP levels are higher in woman than in men (Valdemarsson et al., 1990). There appears to be a greater expression of the CGRP receptors by trigeminal ganglion neurons in male than in female rats, but a higher baseline expression of CGRP in the brainstem of female rats (Stucky et al., 2011). Estrogen does not appear to alter the expression of CGRP in some animal studies, but in others increased its expression (Gupta et al., 2011; Aggarwal et al., 2012). Application of CGRP to the dura of male and female rats resulted in lower facial mechanical withdrawal thresholds in female rats for 72 h, but not in male rats (Avona et al., 2019). Furthermore, dural application of CGRP in female, but not male rats caused nociceptive priming, which was evident up to 14 days post-administration (Avona et al., 2019). Application of CGRP to the dura of male rats does not excite or mechanically sensitize dural afferent fibers, but does increase dural blood flow (Levy et al., 2005). Results from experiments to examine the effect of CGRP on dural afferent fiber response characteristics in female rats have not been reported. However, while CGRP mediates increased blood flow, it does not appear to contribute to the mechanical sensitization of temporalis muscle nociceptors by intramuscular injection of glutamate in female rats, which suggests that CGRP may not directly affect trigeminal nociceptors in either sex (Gazerani et al., 2010). Treatment of ovariectomized rats with estrogen raises CGRP plasma levels and increases CGRP expression in arterioles (Gupta et al., 2011). This is in line with findings that plasma levels of CGRP are higher in women than in men, and increased by estrogen containing birth control pills (Gupta et al., 2011).

##### Glutamate-receptor antagonists

Ketamine is an antagonist at nicotinic, muscarinic and the *N*-methyl-D-aspartate (NMDA) glutamate receptor subtypes. It is a centrally acting analgesic, although it may also exert some of its antimigraine effects by inhibition of neurogenic inflammation, as trigeminal afferent fibers express a subtype of the NMDA receptor (Wang et al., 2012). It is not clear whether ketamine is effective in migraine, although studies with intranasal ketamine suggest that it may reduce headache-related pain (Afridi et al., 2013; Etchison et al., 2018; Benish et al., 2019).

Trigeminal nociceptors in both animals and humans express NR_2__B_ subunit containing NMDA receptors, with a greater expression in females than in males (Wong et al., 2014). The expression of peripheral NMDA receptors by nociceptors in the trigeminal system is modulated by estrogenic tone and NMDA-evoked afferent discharge is also greater in female rats (Dong et al., 2007). In healthy human subjects, injection of glutamate into the masseter muscle results in pain that can be attenuated by co-administration of ketamine. A higher concentration of co-administered ketamine is required to attenuate this muscle pain in women, which is consistent with the greater expression of NMDA receptors by masticatory muscle nociceptors in women (Cairns et al., 2003; Castrillon et al., 2007, 2012). Theoretically, ketamine might be more effective as an analgesic/abortive in men.

##### Opioids

Opioids are occasionally used as abortives and act on three receptor subtypes to exert analgesic effects. These receptors are found throughout the central nervous system and may be expressed by a subgroup of trigeminal primary afferent fibers. Activation of these receptors decreases neurotransmitter release (pre-synaptic inhibition) and neuronal excitability (post-synaptic inhibition). There is some evidence that morphine produces greater analgesia in women and is more likely to be prescribed for women with migraine (Niesters et al., 2010; Connelly et al., 2019).

#### Prophylactic Treatment of Migraine

Prophylactic agents are used to reduce the frequency of migraine attacks and include beta-blockers, calcium channel blockers, antidepressants, botulinum neurotoxin, and antibodies against CGRP. Reports on sexually dimorphic responses to migraine preventive therapy are lacking (Vetvik and Macgregor, 2017). However, it has been found that women are more likely than men to use prophylactic medication (Vetvik and Macgregor, 2017). Levels of prophylactic use in both men and women are far lower than the recommendations (Buse et al., 2013).

Among beta-blockers, propranolol plasma binding has been found similar in both sexes, while its clearance is decreased in women (Soldin et al., 2011). Clearance of oral atenolol is lower in women, which together with its lower volume of distribution results in a higher systemic exposure of women to this agent. Among calcium channel blockers, verapamil clearance is more rapid in women following intravenous administration, whereas after oral administration, clearance is higher in men. The bioavailability of oral verapamil is higher in women, which can lead to an increased systemic exposure in women. Biotransformation of verapamil by CYP3A4 and *p*-glycoprotein (PGP) in liver and gut is different in women and men and can lead to more complex differences in clearance between the sexes (Soldin et al., 2011). Selective serotonin reuptake inhibitors are another class of drugs that are used as prophylactic agents in migraine and overall, due to lower hepatic metabolism, plasma concentrations tend to be higher in women (Soldin et al., 2011). Antibodies against CGRP (e.g., galcanezumab) or its receptor (erenumab) have been marketed recently for prophylactic therapy. It is not yet clear if CGRP antibodies show different efficacy in women and men. However, erenumab, when it was tested in a rat model of IS, showed no sex-related difference in efficacy (Stucky et al., 2011).

### Potential Mechanisms of Sex-Related Responses to Pharmacotherapy of Migraine

**FIGURE 1:**
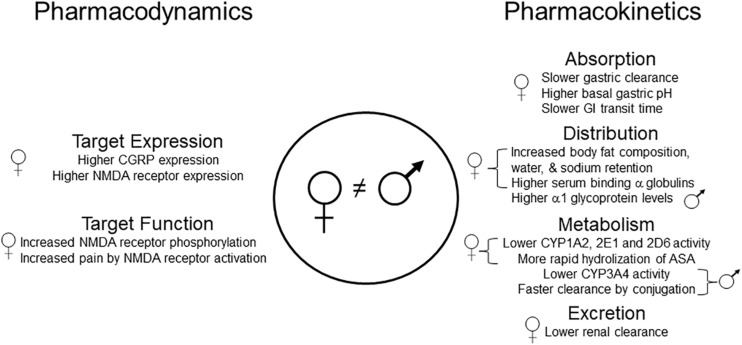
Pharmacodynamic and pharmacokinetic mechanisms that could, theoretically, differentially affect the disposition of drugs used for migraine therapy in men and women. Specific examples include lower levels of ASA in women due to more rapid absorption and degradation, higher levels of propranolol, codeine, and tramadol in women due to lower CYP activity, and lower levels of paracetamol in men due to more rapid glucuronidation. It remains unknown whether any of these sex-related differences contribute to the effectiveness of these or other analgesic drugs when used for migraine treatment in men and women. ASA, acetylsalicylic acid; CYP, cytochrome P450; CGRP, calcitonin gene-related peptide; GI, gastrointestinal; NMDA, *N*-methyl-D-aspartate.

There is limited information about underlying mechanisms of sex-related differences in the pharmacotherapy of migraine. The studies reviewed here suggest that both pharmacodynamic (PD) and PK mechanisms may underlie sex-related differences in response to migraine pharmacotherapy (Figure 1). Expression of CGRP and its receptors appears to be different in males and females, which may suggest PD-related mechanisms. In this respect, it might be proposed that women would be better responders to abortive and/or prophylactic therapy because there is a stronger relationship between CGRP expression and migraine headache in women. Sex-related differences in absorption, distribution, metabolism, and excretion of therapeutic agents for migraine may result in PK-related mechanisms. For example, ingested oral ASA is more rapidly eliminated in women than in men, which may explain an apparent greater analgesic efficacy of ASA in men.

For future investigation in both preclinical and clinical trials, it is recommended that the study design includes the question of potential sex-related differences in therapeutic response and their mechanism (Vetvik and Macgregor, 2017). Another important aspect is the current lack of information on sex-differences in the adverse effects of analgesic drugs in the literature (Richardson and Holdcroft, 2009). For example, unwanted effects following administration of opioid; including nausea, vomiting, and respiratory depression occur more often in women than in men (Fillingim et al., 2005). In this context, sex-related differences in metabolism of certain analgesics, such as acetaminophen and ASA, as well as contributions from sex hormones, genetic variation, and co-administration of drugs for comorbid conditions, such as depression, may differentially affect adverse response to analgesics. It has also been proposed that women have a greater awareness of medication risks, which may be another factor that contributes to sex-related differences in the occurrence of certain adverse effects (Richardson and Holdcroft, 2009).

As has already been found in neuropathic pain, future research should also clarify if non-neuronal cells, e.g., glia, play a role in sex-related differences in migraine and its treatment (Machelska and Celik, 2016).

Since psychosocial factors, such as expectations, stereotypes, cultural differences, pain-related beliefs, past experiences of pain, and environmental stress can influence pain perception (Packiasabapathy and Sadhasivam, 2018), it is highly valuable to consider how these factors may influence sex-related differences in response to migraine pharmacotherapy. Women use a diverse range of pain coping strategies including social support, emotion-focused techniques, attention focus, cognitive re-interpretation, and positive self-statement (Unruh et al., 1999; Packiasabapathy and Sadhasivam, 2018). On the other hand, problem-focused techniques and behavioral distraction are more common strategies used by men (Unruh et al., 1999; Fillingim et al., 2009; Packiasabapathy and Sadhasivam, 2018). Catastrophizing with magnification and self-rumination of pain is higher in women (Forsythe et al., 2011). Taken together, these factors reinforce the value of recognition and utilization of the biopsychosocial model (Engel, 1977; Borrell-Carrio et al., 2004) in migraine research and treatment. The influence of psychological interventions, such as behavioral modifications, or pain coping strategies, on response to migraine pharmacotherapy needs to be considered in future studies.

## Author Contributions

PG and BC contributed equally to the writing of this narrative review and agreed on the final version.

## Conflict of Interest

The authors declare that the research was conducted in the absence of any commercial or financial relationships that could be construed as a potential conflict of interest.
